# Decreased Memory B Cells and Increased CD8 Memory T Cells in Blood of Breastfed Children: The Generation R Study

**DOI:** 10.1371/journal.pone.0126019

**Published:** 2015-05-18

**Authors:** Michelle A. E. Jansen, Diana van den Heuvel, Menno C. van Zelm, Vincent W. V. Jaddoe, Albert Hofman, Johan C. de Jongste, Herbert Hooijkaas, Henriette A. Moll

**Affiliations:** 1 The Generation R Study Group, Erasmus MC, University Medical Center, Rotterdam, The Netherlands; 2 The Department of Pediatrics, Sophia Children’s Hospital, Erasmus MC, University Medical Center, Rotterdam, The Netherlands; 3 The Department of Immunology, Erasmus MC, University Medical Center, Rotterdam, The Netherlands; 4 The Department of Epidemiology, Erasmus MC, University Medical Center, Rotterdam, The Netherlands; INRA, UR1282, FRANCE

## Abstract

**Background:**

Breastfeeding provides a protective effect against infectious diseases in infancy. Still, immunological evidence for enhanced adaptive immunity in breastfed children remains inconclusive.

**Objective:**

To determine whether breastfeeding affects B- and T-cell memory in the first years of life.

**Methods:**

We performed immunophenotypic analysis on blood samples within a population-based prospective cohort study. Participants included children at 6 months (n=258), 14 months (n=166), 25 months (n=112) and 6 years of age (n=332) with both data on breastfeeding and blood lymphocytes. Total B- and T-cell numbers and their memory subsets were determined with 6-color flow cytometry. Mothers completed questionnaires on breastfeeding when their children were aged 2, 6, and 12 months. Multiple linear regression models with adjustments for potential confounders were performed.

**Results:**

Per month continuation of breastfeeding, a 3% (95% CI -6, -1) decrease in CD27+IgM+, a 2% (95 CI % -5, -1) decrease in CD27+IgA+ and a 2% (95% CI -4, -1) decrease in CD27-IgG+ memory B cell numbers were observed at 6 months of age. CD8 T-cell numbers at 6 months of age were 20% (95% CI 3, 37) higher in breastfed than in non-breastfed infants. This was mainly found for central memory CD8 T cells and associated with exposure to breast milk, rather than duration. The same trend was observed at 14 months, but associations disappeared at older ages.

**Conclusions:**

Longer breastfeeding is associated with increased CD8 T-cell memory, but not B-cell memory numbers in the first 6 months of life. This transient skewing towards T cell memory might contribute to the protective effect against infectious diseases in infancy.

## Introduction

Breast milk contains factors that enhance nutrient absorption, stimulate growth and enhance the defense against pathogens [1]. Consequently, breastfeeding provides protection against infectious diseases during infancy [2,3,4]. The protective effect persists during childhood [5,6], and modulates vaccination responses [7,8,9]. Thus, it is likely that breastfeeding not only provides passive immunization, but also enhances adaptive immunity [10].

B and T lymphocytes comprise the cellular components of adaptive immunity, and are generated throughout life. B cells contribute to humoral immunity through the production of immunoglobulins (Ig), whereas CD8+ cytotoxic T cells provide cellular immune responses. CD4+ helper T cells support both humoral and cellular immune responses. Each B and T cell generates a unique antigen receptor during precursor differentiation in bone marrow or thymus, respectively. Only those cells that specifically recognize antigen with their receptor will undergo clonal proliferation and are involved in the antigen response. Cells generated from the clonal expansion will remain present in the body as long-lived memory cells and will initiate a fast and quantitatively stronger response upon secondary antigen encounter. In addition to CD27- naive B cells, six memory B-cell subsets can be identified [11]. Four of these express CD27 and are either positive for IgM, IgM and IgD, IgA or IgG. In addition, CD27-IgA+ and CD27-IgG+ memory B cells can be identified. Within both the CD4 and CD8 T-cell lineages, central memory (CD45RO+CCR7+), CD45RO+CCR7- effector memory (TemRO) and CD45RO-CCR7- (TemRA) can be distinguished from naive T cells (CD45RO-CCR7+) [12]. Central memory T cells are most efficient in generating a new immune response by proliferating extensively in response to an antigen upon secondary antigen encounter [13,14,15]. The diversity and composition of the B- and T-cell compartments are highly dynamic in the first years of life; blood cell counts are especially high up to 2 years of age, following which they slowly decline to reach adult levels between 6 and 10 years [16,17,18,19,20]. At birth, nearly all B and T cells are naive, and memory cells are gradually built up in the first 6 years of life. [19].

Several studies have addressed the effects of breastfeeding on adaptive immunity. Breastfeeding was found to be associated with decreased frequencies of blood CD4+ T cells. [21] This was mostly due to lower frequencies of naïve (CD45RA+) T cells in breastfed children. Still, these observations were not consistently reproduced with some studies showing increased, and some decreased numbers of CD4+ T cells. [21,22,23,24] Furthermore, long term breastfeeding was found to be associated with increasing numbers of CD4+ and CD8+ T cells.[24] Thus, although previous studies have addressed the influence of breastfeeding on blood lymphocyte populations [21,22,23,24], the results remained inconclusive, mainly due to small samples sizes and limitations in the detection of memory cells [21,22,23,24].

In the present study we used 6-color flow cytometric analysis of lymphocyte subsets in a population-based prospective cohort study to assess the impact of breastfeeding on build-up of memory B and T cells in infants and young children.

## Materials and Methods

### Design and study population

This study was embedded in the Generation R Study, a prospective population-based cohort study that follows pregnant women and their children from fetal life onwards in the Netherlands [25]. The study has been approved by the Medical Ethics Committee of the Erasmus MC, University Medical Centre Rotterdam. Written informed consent was obtained from all parents of participants. We included 1,079 Dutch pregnant women and their children participating in a detailed subgroup study [25]. All children were born between February 2002 and August 2006. We excluded twins (n = 27) in the present analysis to prevent bias due to correlation. Of these, data on both breastfeeding and immunophenotyping were available from 258 children at 6 months, 166 at 14 months, 112 at 25 months and 332 at 6 years of age. The main reasons for missing samples were due to non-consent of the parents (approximately 55% per visit) and technical or logistical failure (approximately 10% per visit).

### Breastfeeding

Information regarding breastfeeding was obtained in postnatal questionnaires at the ages of 2, 6 and 12 months [25]. Mothers were asked whether they had ever breastfed their child (yes or no) and, if yes, at what age (months) they had stopped [26,27]. Breastfeeding duration was then categorized into four groups: never, ≤3 months, between 3 and 6 months and ≥6 months. An approximation of exclusive breastfeeding was performed according to whether the child received breastfeeding without any other bottle feeding, milk or solids [27]. Partial breastfeeding indicates infants receiving both breast-feeding, bottle feeding and/or solids in this period. Subsequently, the information on exclusiveness of breastfeeding was combined and categorized into the following breastfeeding categories: never; partial until 4 months and exclusive until 4 months.

### Immunophenotyping of lymphocyte subsets

Flow cytometry was performed within 24 hours following sampling on fresh whole blood at the ages of 6 months (median 6.2; range 5.2; 8.2), 14 months (median 14.4; range 13.1–17.4), 25 months (median 25.2; range 23.3–29.8) and 6 years (median 5.9; range 5.1–7.2). Absolute counts of blood CD3+ T cells, CD16/56+ NK cells, and CD19+ B cells were obtained with a diagnostic lyse-no-wash protocol. Lymphocytes were gated on the basis of CD45, FSC and SSC characteristics. Gates were set based on cells that are known to lack expression of the indicated marker. Additionally, 6-color flow cytometry was performed on an LSRII (BD Biosciences) to distinguish naive and memory B- and T- lymphocyte subsets as defined previously (S3 Table) [11,12]. All flow cytometry acquisition was performed on whole blood after red blood cell lysis with ammonium chloride.

### Covariates

The covariates that were assessed in this study were obtained from midwife and hospital registries at birth (birth weight, gestational age and gender) or through measurements at the research center (child anthropometrics). Additionally, information on smoking and alcohol use during pregnancy and socioeconomic status was obtained by prenatal questionnaires sent during the first, second and third trimesters of pregnancy [28 29]. Information on day-care attendance was obtained from parent-reported questionnaires at the ages of 6 and 12 months.

### Statistical methods

Because the distribution of lymphocyte numbers in different age groups was skewed, these values were normalized by transformation to a natural log-scale. Differences in maternal and infant characteristics between breastfed versus never breastfed children were tested using independent samples t-tests and Chi-Square tests. Differences in baseline characteristics among the groups with different duration of breastfeeding were assessed using ANOVA tests and Chi-Square tests. Additionally, the associations of breastfeeding, breastfeeding duration (measured in groups, and measured continuously per month continuation of breastfeeding) and breastfeeding exclusivity with the change in lymphocyte numbers were assessed using multiple linear regression models with adjustment for potential confounders. For all ages, the category with no breastfeeding was the reference. First, associations between breastfeeding and total B, T, CD4 and CD8 counts were assessed. Subsequently, associations for B, CD4 and CD8 subpopulations were studied to assess the effect of breastfeeding on memory cells specifically. Multivariable regression models were created with stepwise adjustment for potential confounders, which were selected based on previous literature. Potential confounders included: maternal age, socioeconomic status (SES), marital status, maternal BMI, maternal smoking and alcohol consumption during pregnancy, maternal reported autoimmune disease (including thyroid disease, multiple sclerosis, systemic lupus erythematosus, diabetes mellitus and arthritis), elevated anti-tTG level during pregnancy, maternal fever in the last trimester of pregnancy, family history of asthma or atopy (hay-fever, allergy, eczema), multiple parities, mode of delivery (caesarean section), gender, birth weight, gestational age, preterm birth, APGAR score, birth season, weight and age at focus visit, fever in the first 6 months (yes/no), frequency of upper and lower respiratory tract infections, and day-care attendance in the first year of life. Covariates were kept in the final multivariate model if the covariate resulted in an alteration in effect estimate of ≥ 10% [30], or if the variables were associated with breastfeeding (determinant) and lymphocyte numbers (outcome) in our study. Because of the small numbers in the never breastfed group, final adjustment for potential confounders was restricted to those who attained the strongest alteration (%) in effect estimates. Because of the strong correlation between our outcomes (e.g. Pearson’s correlation between total CD8 T cells and naive CD8 T cells r = 0.82, and between total B cells and IgA r = 0.57), and unweighted calculations only hold if the tests are independent [31,32], we did not perform adjustments for multiple testing. All measures of associations are presented with their 95% confidence interval. All statistical analyses were performed using the Statistical Package for the Social Sciences version 20.0 for Windows (SPSS Inc, Chicago, IL, USA). P values <0.05 were considered to be statistically significant.

## Results

### Population characteristics

No major differences in characteristics between children included at 6, 14, 25 months and 6 years of age were observed (Table 1, S1 Table) Overall, more than 86% of mothers started breastfeeding. Mother’s educational level was significantly associated with the start of breastfeeding at 6 and 25 months, and 6 years of age. Moreover, mother’s educational level was significantly associated with both the duration of breastfeeding and with B cell memory subsets at 6 months of age [data not shown]. In addition, maternal alcohol use was related to both breastfeeding duration and total T, B, CD4 and CD8 cell numbers at 6 months [data not shown]. Both maternal educational level and alcohol use influenced the regression coefficients by more than 10%. Therefore, all subsequent analysis on breastfeeding duration and cell numbers were adjusted for both maternal education and maternal alcohol use during pregnancy.

**Table 1:** Maternal and infant characteristics of the study population at 6 months.

	Not breastfed (n = 35)	Breastfed (n = 223)
**Maternal characteristics (n = 258)**		
Age (Mean ± SD; years)	32 (3.6)	32 (3.8)
Educational level (n; %)		
Lower	24 (69%)	71 (32%)
Higher	11 (31%)	152 (68%)*
Net household income per month (n; %)		
< € 2400	2 (7%)	23 (11%)
≥ € 2400	29 (93%)	178 (89%)
Smoking continued during pregnancy (n; %)	4 (14%)	21 (13%)
Alcohol use continued during pregnancy (n; %)	8 (29%)	55 (35%)
Body Mass Index before pregnancy (Mean ± SD; kg/m^2^)	23 (3)	23 (4)
Fever in third trimester of pregnancy (n; %)	3 (9)	14 (6%)
Maternal atopy (eczema, allergy HDM, hay-fever)(n; %)	8 (25%)	72 (35%)
Paternal atopy (eczema, allergy HDM, hay-fever) (n; %)	8 (28%)	55 (28%)
Family history of asthma / atopy (n; %)	13 (37%)	107 (49%)
Any reported autoimmune disease (diabetes mellitus, SLE, arthritis, MS, thyroid disorder, or celiac disease) (n; %)	0 (0%)	5 (0.02%)
Mode of delivery (n; %)		
Vaginal	21 (64%)	129 (61%)
Forceps or vacuum assisted	5 (15%)	44 (20%)
Caesarian section	7 (21%)	40 (19%)
Premature rupture of membranes (n; %)	3 (9%)	6 (3%)
**Infant characteristics (n = 258)**		
Males (n; %)	19 (54%)	113 (51%)
Gestational age (Mean ± SD; weeks)	39.4 (2.4)	40.0 (1.7)
Preterm birth (<37 weeks) (n; %)	2 (6%)	11 (5%)
Birth weight (Mean ± SD; grams)	3439 (632)	3504 (524)
Apgar score at 5 min <7 (n; %)	0 (0%)	3 (1%)
Birth season (n; %)		
Winter (dec-jan-feb)	5 (14%)	36 (16%)
Spring (mar-apr-may)	10 (29%)	81 (36%)
Summer (jun-jul-aug)	11 (31%)	62 (28%)
Autumn (sept-oct-nov)	9 (26%)	44 (20%)
Siblings ≥1 (n; %)	4 (11%)	26 (12%)
Day-care >16 hours /week (n; %)	5 (33%)	77 (46%)
Fever in first 6 months (n; %)	12 (60%)	121 (62%)
Age at focus visit (Median ± range; months)	6.6 (6.2–8.2)	6.2 (5.2–7.9)*

Values are means (SD), absolute numbers (percentages) or ^#^medians (90% range).

*Significantly different between groups.

Data were missing on: Household income (n = 26) Smoking during pregnancy (*n* = 72), Alcohol during pregnancy (*n* = 72) BMI before pregnancy (n = 37), Fever in third trimester (n = 9) Mode of delivery (n = 12) Maternal atopy (n = 22) Paternal atopy (n = 29), Family history of asthma or atopy (n = 3) Mode of delivery (n = 12), maternal reported autoimmune disease (n = 29), maternal reported any other chronic condition (n = 31), premature rupture of membranes (n = 5), Apgar (n = 4), Day-care (n = 42), weight at focus visit (n = 1), fever in first 6 months (n = 42). Any reported autoimmune disease included thyroid disease (n = 4) and elevated anti-tTG level during pregnancy (n = 1).

### Decrease in B-cell memory

**Fig 1:**
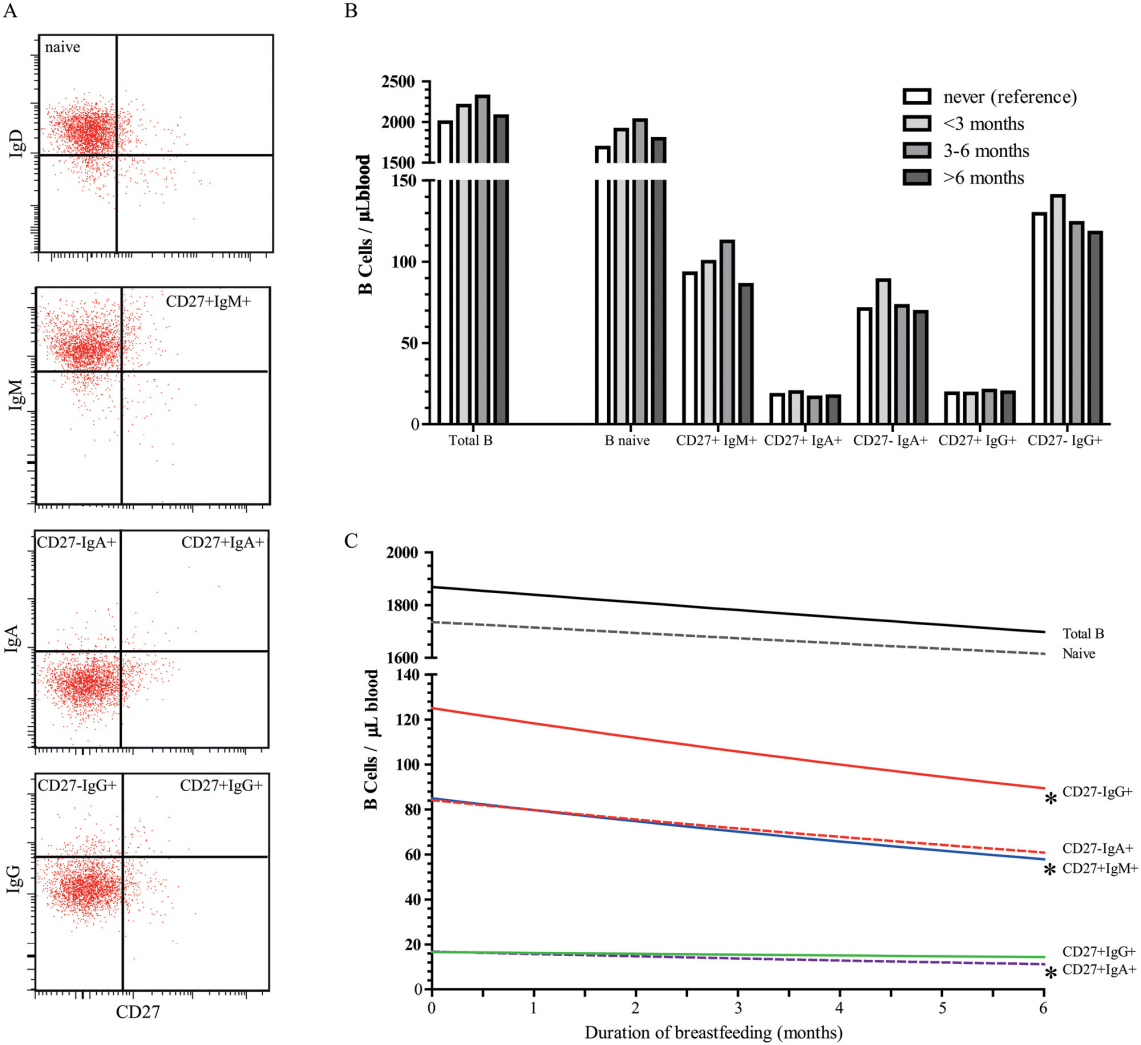
Impact of breastfeeding duration on B lymphocyte subsets at 6 months of age. **A,** Gating strategy for dissection of CD19+ B cells into 1 naive and 5 memory B-cell subsets by flow cytometry. The reference plots depict density plots of total lymphocytes and were used to set the gates accordingly. **B,** Backtransformed B-cell counts (cells/μL) at 6 months of age according to different breastfeeding duration categories (reference category is never). Categories of breastfed children contain both partial and exclusively breasted children. **C,** The estimated backtransformed regression line reflects B-cell counts (cells/μL) at 6 months of age, per month increase in breastfeeding duration.*, P<0.05.

Breastfeeding exposure (Fig 1b) and duration (Fig 1c) were not associated with total B cell numbers at 6, 14, 25 months and 6 years of age (Table 2, S2 Table). Furthermore, no associations were observed between breastfeeding exposure and duration and naive B cells, which constitute the majority of total B-cell numbers (Table 3, Fig 1b and 1c) However, a longer duration of breastfeeding, was associated with changes in the memory B-cell compartment. Per month longer breastfeeding, a 3% decrease in absolute numbers of CD27+IgM+, and a 2% decrease in both CD27+IgA+ and CD27-IgG+ memory B cells at 6 months of age were observed (Table 3, Fig 1c). CD27+IgG+ and CD27-IgA+ memory B cells at 6 months of age did not change with differences in breastfeeding duration. Stronger negative trends were observed for the associations between breastfeeding duration and frequencies of CD27+IgM+, CD27+IgA+ and CD27-IgG+ memory B cells within total B cells at 6 months of age, resp. -4% (95% CI -7, -1), -4% (95% CI -7, -1), and -3% (95% CI -5, -1) (data not shown). At 14 months of age, similar trends for breastfeeding duration and total B-cell numbers were observed, although not significant (Table 2). At older ages the effects disappeared. (S2 Table). Thus, a longer breastfeeding duration seems to negatively impact B-cell numbers in infants at least until the age of 6 months, at older ages these effects disappeared.

**Table 2:** Adjusted associations between breastfeeding and cell numbers at 6 and 14 months.

	Regression coefficients for logtransformed cell numbers (*10e9/L)										
	6 months (n = 258)					14 months (n = 166)					
	B	NK	T	CD4	CD8		B	NK	T	CD4	CD8
**DURATION**						**DURATION**					
Breastfeeding (n = 223) increase per month	-1(-3,1)	0 (-2,2)	-1 (-2,1)	1(-2,1)	1 (-1,2)	Breastfeeding(n = 150) increase per month	-2 (-4,0)	0 (-2,2)	0 (-2,1)	0 (-2,1)	0 (-2,2)
**EXPOSURE**						**EXPOSURE**					
Never (n = 35)	REF	REF	REF	REF	REF	Never (n = 16)	REF	REF	REF	REF	REF
< 3 months (n = 86)	9 (-8,25)	4 (-16,24)	7 (-5,19)	6 (-7,19)	**19 (3,35)** *	< 3 months (n = 55)	15 (10,40)	21 (-5,47)	16(-3,35)	13 (-6,32)	19 (-5,44)
≥ 3 < 6 months (n = 64)	6 (-12,23)	0 (-21,22)	8 (-5,21)	7 (-7,22)	**20 (3,37)** *	≥ 3 < 6 months (n = 40)	26 (0,52)	-2 (-28,25)	20 (0,39)	18 (-2,38)	25 (0,50)
≥ 6 months (n = 73)	0 (-18,18)	9 (-13,31)	2 (-11,16)	0 (-14,15)	16 (-2,33)	≥ 6 months (n = 55)	2 (-22,27)	9 (-16,34)	13 (-5,31)	13 (-6,31)	13 (-11,36)

Values are log transformed regression coefficients (95% confidence interval of cell numbers (*10^e9^/L)) derived from linear regression models and reflect the increase or decrease (%) in log transformed cell numbers per month continuation of breastfeeding.(duration), and the increase or decrease (%) in log transformed cell numbers of groups exposed to breastfeeding relative to the reference group (never breastfeeding). Breastfed groups include both partially and exclusively breastfed children.

*P-value <0.05. Adjusted for maternal education and maternal alcohol use during pregnancy.

**Table 3:** Adjusted associations between breastfeeding and memory B- and T-cell subsets at 6 months.

	Regression coefficients for logtransformed cell numbers (*10e9/L)													
	B (n = 258)						T CD4 + (n = 258)				T CD8+ (n = 258)			
	Naive B CD27- IgD+	CD27+IgM+	CD27+IgA+	CD27-IgA+	CD27+IgG+	CD27-IgG+	CD4+ T naive	CD4+T cm	CD4+TemRO	CD4+TemRA	CD8+T naive	CD8+T cm	CD8+TemRO	CD8+TemRA
**DURATION**														
**Breastfeeding (n = 223) increase per month**	-1(-3,1)	**-3(-6,-1)** *	**-2 (-5,-1)** *	-2 (-5,1)	1 (-3,2)	**-2 (-4,-1)** *	-2 (-3,0)	-1(-2,1)	-1(-3,2)	0(-3,2)	-2 (-4,1)	0(-2,3)	0(-3,3)	2 (-1,5)
**EXPOSURE**														
**Never (n = 35)**	REF	REF	REF	REF	REF	REF	REF	REF	REF	REF	REF	REF	REF	REF
**< 3 months (n = 86)**	12 (-6,31)	9(-19,36)	1 (-28,30)	15 (-14,43)	-9 (-34,16)	-1 (-22,22)	7(-10,24)	-3(-20,14)	17(-4,38)	14(-8,37)	23 (0,46)	**30 (4,57)** *	15(-18,47)	20(-8,47)
≥ **3 < 6 months (n = 64)**	18 (-1,38)	18(-11,48)	1 (-30,32)	14 (-17,44)	0 (-26,27)	1 (-22,25)	4(-14,22)	-5(-23,13)	9(-14,31)	8(-16,32)	23 (-2,448)	17 (-11,45)	-4(-38,31)	9 (-20,39)
≥ **6 months (n = 73)**	6 (-13,26)	-4(-34,25)	-11 (-40,19)	-2 (-31,28)	-7 (-33,19)	-13 (-36,10)	-5(-23,14)	-3(-21,15)	13(-10,35)	10(-14,34)	10(-15,34)	**29 (1,57)** *	-5(-39,30)	21(-9,51)

Values are log transformed regression coefficients (95% confidence interval of cell numbers (*10^e9^/L)) derived from linear regression models and reflect the increase or decrease (%) in log transformed cell numbers per month continuation of breastfeeding.(duration), and the increase or decrease (%) in log transformed cell numbers of groups exposed to breastfeeding relative to the reference group (never breastfeeding). Breastfed groups include both partially and exclusively breastfed children.

*P-value <0.05. Adjusted for maternal education and maternal alcohol use during pregnancy.

### Increase in T-cell memory

**Fig 2:**
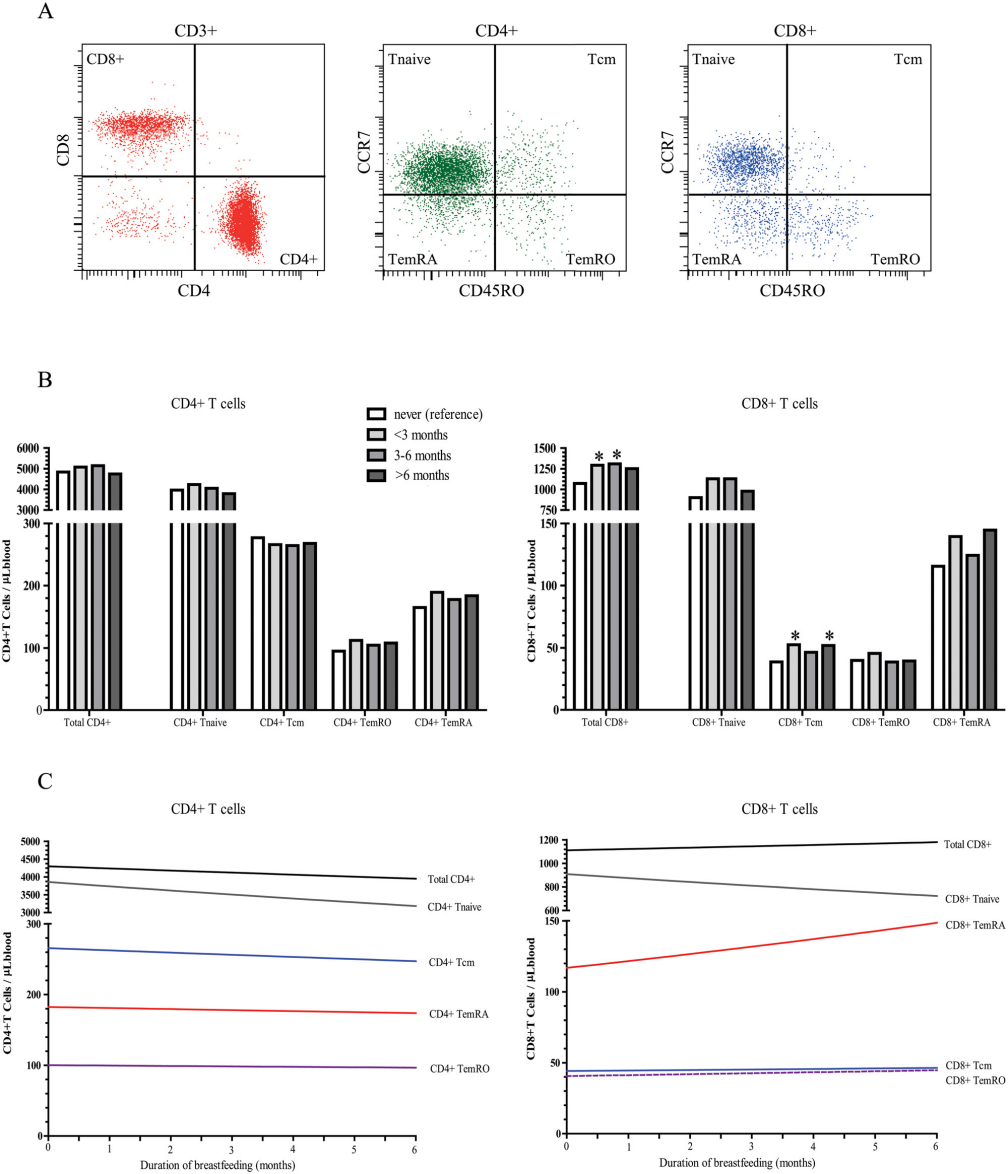
Impact of breastfeeding duration on T-lymphocyte subsets at 6 months of age. **A,** Gating strategy for T-cell subset delineation. CD4+ and CD8+ T-cell subsets were defined within total CD3+ T cells, and subsequently dissected using CD45RO and CCR7 into naive, central memory, CD45RO+ effector memory (TemRO) and CD45RO- effector memory (TemRA) T cells. The reference depict density plots of total lymphocytes and were used to set the gates accordingly. **B,** Backtransformed CD4 and CD8 cell counts (cells/μL) at 6 months of age according to different breastfeeding duration categories (reference category is never). Categories of breastfed children contain both partial and exclusively breasted children. **C,** The estimated backtransformed regression line reflects CD4 and CD8 cell counts (cells/μL) at 6 months of age, per month increase in breastfeeding duration. *, P<0.05.

Breastfeeding exposure and duration were not associated with total T-cell and CD4+ T-cell numbers at 6, 14, 25 months and 6 years of age (Table 2, Fig 2b and 2c, and S2 Table). However, the exposure to breastfeeding was associated with CD8+T cell numbers. CD8+ T-cell numbers were 19% (95% CI 3, 35) higher in 6-month old infants who were breastfed for less than 3 months and remained 20% (95% CI 3, 37) higher in children who were breastfed until 6 months (Table 2, Fig 2b), than in children who were never breastfed. Comparable effect sizes were observed for exclusiveness of breastfeeding in relation to CD8 T cells (Tables 4 and 5). Partial breastfeeding until 4 months was associated with a 20% increase in total CD8 T cells, and remained 21% higher in infants who were exclusively breastfed (Table 5). At 14 and 25 months of age, similar tendencies for breastfeeding exposure and CD8+ T cell numbers were observed, however the associations were not significant. (Table 2, S2 Table).

**Table 4:** Unadjusted associations between breastfeeding exclusivity and CD8+ T cell numbers at 6 months.

Regression coefficients for logtransformed cell numbers (*10e9/L)					
6 months (n = 258)					
	CD8 Total	CD8 Tnaive	CD8 Tcm	CD8 TemRA	CD8 TemRO
**EXCLUSIVENESS**					
**Never (n = 35)**	REF	REF	REF	REF	REF
**Partial until 4 months (n = 157)**	13 (-3,29)	9 (-11,29)	17 (-7,41)	-5 (-30,21)	3 (-27,32)
**Exclusive until 4 months(n = 80**	14 (-3,31)	3 (-19,25)	**33 (7,59)** *	12 (-16,40)	28 (-3,59)

Unadjusted values are log transformed regression coefficients (95% confidence interval of cell numbers (*10^e9^/L)) derived from linear regression models and reflect the increase or decrease (%) in log transformed cell numbers of partial and exclusive breastfed groups relative to the reference group (never breastfeeding). Partial breastfeeding indicates infants receiving both breast-feeding, bottle feeding and/or solids in this period.

*P-value <0.05.

**Table 5:** Adjusted associations between breastfeeding exclusivity and CD8+T cell numbers at 6 months.

Regression coefficients for logtransformed cell numbers (*10e9/L)					
6 months (n = 258)					
	CD8 Total	CD8 Tnaive	CD8 Tcm	CD8 TemRA	CD8 TemRO
**EXCLUSIVENESS**					
**Never (n = 35)**	REF	REF	REF	REF	REF
**Partial until 4 months (n = 143)**	**20 (3, 38)** *	20 (-5, 44)	12 (-17,40)	18 (-11, 47)	4 (-31, 39)
**Exclusive until 4 months (80)**	**21 (2, 40)** *	14 (-14, 41)	16 (-15, 48)	27 (-5, 59)	12 (-28, 51)

Values are log transformed regression coefficients (95% confidence interval of cell numbers (*10^e9^/L)) derived from linear regression models and reflect the increase or decrease (%) in log transformed cell numbers of partial and exclusive breastfed groups relative to the reference group (never breastfeeding). Partial breastfeeding indicates infants receiving both breast-feeding, bottle feeding and/or solids in this period.

*P-value <0.05. Adjusted for maternal education and maternal alcohol use during pregnancy.

Within the CD8 T-cell compartment at 6 months of age, central memory cell numbers (CD45RO+CCR7+) were 30% (95% CI 4, 57) higher in children who were breastfed until 3 months than in non-breastfed children (Table 3, Fig 2b). This effect was irrespective of duration; central memory cell numbers were still 29% (95% CI 1, 57) higher in infants who were breastfed for 6 months or longer than in infants who were never breastfed (Table 3, Fig 2b). In line with this, no significant associations were found between the duration of breastfeeding and CD8 central memory numbers (Table 3, Fig 2c).

Thus, CD8 central memory T cells were associated with breastfeeding exposure, but not with the duration of breastfeeding.

## Discussion

Overall we found that breastfeeding was associated with a decrease in B-cell memory and an increase in CD8 T-cell memory at 6 months of age. The same trend was observed at 14 months of age, and disappeared at older ages. The decrease in B-cell memory was associated with the duration of breastfeeding, while the increase in total CD8 T cells and central memory CD8 T cells did not depend on the duration of breastfeeding. This suggests that breastfeeding enhances T-cell maturation, but not B-cell maturation.

Total B-cell numbers nor frequencies were significantly related with breastfeeding. This is in line with previous studies [21,23]. Only one study reported higher frequencies of total B cells in breastfed children at 6 months of age, however sample sizes were small (n = 7 breastfed infants)[22]. We found that the duration of breastfeeding was associated with decreased numbers of CD27+IgA+, CD27+IgM+ and CD27-IgG+ memory B cells, which are mostly derived from systemic T-cell dependent responses [11]. This suggests that continuous breastfeeding inhibits memory B-cell development. One of the candidate factors in breast milk that is likely to mediate this, is secretory IgA (sIgA) [33,34]. Continuous breastfeeding will provide a constant supply of maternal sIgA onto the epithelial surface of the infant’s gastrointestinal tract. This sIgA might prevent exposure of microorganisms to the infant’s humoral immune system, and translocation of gut bacteria [34,35]. Thus, fewer naive B cells might be activated to differentiate into memory B cells (S1 Fig). Indeed, in suckling mice it has been suggested that maternal sIgA blocks mucosal B cell responses in the offspring [36,37]. Alternatively, the unchanged numbers of T-cell independent CD27-IgA+ B cells suggest that breastfeeding does not affect local IgA responses. It is therefore possible that maternal IgA helps to block translocation of intestinal bacteria, thereby only preventing systemic T-cell dependent B-cell memory formation. Other growth factors in breast milk that can reduce exposure of microbiota to B cells are epidermal growth factor, IGF-1, TGF-β, leptin and prolactin. These factors enhance maturation of the epithelial barrier [33,38], and decrease uptake of foreign protein antigens. Factors such as lactoferrin, oligosaccharides and lipids may directly prevent attachment of the bacterial outer membrane to the mucosal surface [33]. Finally, the passive transfer of functional Ig-secreting plasma cells in breast milk may prevent bacterial or viral transmission [39,40].

Total T-cell numbers nor CD4 T-cell numbers were associated with breastfeeding exposure or breastfeeding duration. However, infants who were breastfed until 3 months had higher CD8 T-cell numbers than infants who were never breastfed, and this change persisted when breastfeeding was prolonged until 6 months, suggesting an ongoing activation of CD8 T cells by exposure to breast milk, but no accumulation over time. These results extend previous observations in small studies (n<40) of increased frequencies of CD8 T cells or decreased CD4/CD8 ratios [21,22,24]. In addition, an increase in CD8 T cells from 8 to 10 months of age was observed before [24], suggesting a longer-lasting effect of breastfeeding. However, the children received breastfeeding until 8 months of age [24]. In addition, the number of children in the breastfeeding group was small (n = 35), and no detailed analysis of CD8 subsets was performed. Therefore, future studies will be needed to validate these findings.

Within total CD8 T cells, the central memory subset was most significantly increased in breastfed children. This expansion will be the result of stimulation of mature T cells. Candidate immune stimulatory factors in breast milk are lactoferrin and exosomes. Lactoferrin has direct microbicidal properties, including binding of the bacterial cell wall and the concomitant release of lipopolysaccharide (LPS, endotoxin). Moreover, lactoferrin is known to enhance T-cell proliferation [21,33]. Exosomes are carrier vesicles of 50–100 nm that can bud from the membrane of eukaryotic cells [41]. Exosomes formed by B cells or dendritic cells contain MHC class I and class II, and have the potential to stimulate T cells [42,43]. Human breast milk has been found to contain immune modulatory exosomes that express MHC molecules, IL-2, IFNγ and TNFα. In line with our findings that breastfeeding did not affect memory CD4 T cells, these vesicles did not appear to stimulate CD4 T cells [44]. It is, however, likely that immunostimulatory compounds such as exosomes, do have CD8 T-cell stimulating capacities and contribute to the increase in central memory T cells in breastfed infants (S1 Fig). In addition, it has been described that the infants take up live and functional maternal immune cells from the breast milk [45,46,47]. This might result in stimulation of CD8 T cells. Thus, it remains unclear whether the increase in central memory CD8 T cells can be attributed to immunostimulatory factors in breast milk, such as lactoferrin and exosomes, or reflects a role for maternal immune cells. Nevertheless, central memory T cells might mediate reactive immunity [48], because they circulate between the spleen, blood and lymph nodes and proliferate extensively in response to a second encounter of an antigen [13,14,15]. Therefore, it could be hypothesized that an increase in central memory T cells is associated with increased reactive immunity.

Beneficial effects of breastfeeding on adaptive immune responses have previously been demonstrated in vaccination studies [7,8,9]. Breasted children showed increased interferon-γ production, and increased frequencies of CD8+ T cells after vaccination with measles, mumps and rubella [9]. Furthermore, breastfeeding had beneficial effects on virus-specific immune responses to poliovirus, diphtheria toxoid and tetanus toxoid [49], whereas the responses to rotavirus are not clearly enhanced [50,51]. These studies included relatively few children, and confounding factors could not be taken into account. Thus, our results extend these previous observations from vaccination studies. Unfortunately, we were not able to study virus-specific memory cells and studies determining associations between central memory cell numbers and functional immunity are lacking. Thus, interpretations regarding cell-mediated immunity should be made with caution.

We did not observe associations between breastfeeding and naive T cells or effector memory T cells. In contrast, a previous study reported lower frequencies of naive CD4 T cells [21], but no differences in memory T-cell frequencies. Therefore, it was suggested that the adaptive immune system develops slower in breastfed infants [21]. However, the study reported on a relatively small number of children in the breastfeeding group (n = 34), and lacked data on absolute B- and T-cell counts [21].

Our study was conducted in a large population-based prospective birth cohort. Previous studies that addressed the effects of breastfeeding on adaptive immunity, had smaller sample size and/or lacked follow-up [21,22,23,24]. Most of these retrospective studies were based on recall of infant feeding after several years, making recall bias of feeding habits an important concern [52]. Because of the prospective design of our study, detailed information on the duration of breastfeeding was collected at multiple time points shortly after breastfeeding was finished, thereby limiting potential recall bias [52]. Still, data on breastfeeding was collected retrospectively, and due to the use of questionnaires, misclassification may occur [52]. Because any misclassification would be independent of laboratory determined T and B cell numbers, it is unlikely that these affected the outcome. In addition, our study design provided information on a large number of potential confounders. We used an unbiased approach, investigating a broad panel of determinants on lymphocyte numbers and frequencies. Moreover, we performed measurements of lymphocytes at different ages, enabling us to study the effect of breastfeeding on adaptive immunity over a longer period of time. Finally, using detailed 6-color flow cytometry, we were able to discriminate multiple, functionally distinct B- and T-cell subsets [11,12,53]. Thus, we could evaluate the effects of breastfeeding on specific aspects of adaptive immunity.

At 14 months or age, we observed the same trends of decreasing B-cell numbers with longer breastfeeding duration, and higher CD8 T-cell numbers with breastfeeding exposure, as we did for 6 months, although effects were not significant. The sample size at 14 months was smaller than the sample size at 6 months. Hence, we cannot exclude that non-significant effects at 14 months are due to loss of statistical power, and some effects of breastfeeding remain at this age.

Our measurements were limited to peripheral blood. Thus, it is not possible to deduce whether B- and T-cell numbers in lymphoid tissue were affected. Theoretically, preferential homing to or away from tissue could result in changes in blood lymphocyte counts [54]. If breastfeeding affects this preferential homing, the effects will be lost once breastfeeding is discontinued. Nearly all children were no longer receiving breast milk and still showed lower memory B cell numbers and higher CD8 T-cell numbers. Thus, we conclude that the effects of breastfeeding on preferential tissue homing was limited.

In conclusion, this prospective population-based cohort study among a large number of healthy children showed that CD27+IgA+, CD27+IgM+ and CD27-IgG+ memory B-cell numbers decreased with a longer breastfeeding duration. CD8 T cells, and especially CD8 central memory T-cell numbers, were higher in breastfed children up to 6 months of age. This suggests that breastfeeding enhances T-cell maturation in the first 6 months of life. On top of the protective effects of maternal IgA in breast milk, this might contribute to the protective effect against infectious diseases in infancy.

## Supporting Information

S1 Table Maternal and infant characteristics of the study population at 14, 25 months and 6 years. Values are means (SD), absolute numbers (percentages) or ^#^medians (90% range). * Significantly different between breastfeeding and no breastfeeding groups.
(DOC)

S2 Table Adjusted associations between breastfeeding and cell numbers at 25 months and 6 years. Values are log transformed regression coefficients (95% confidence interval of cell numbers (*10^e9^/L)) derived from linear regression models and reflect the increase or decrease (%) in log transformed cell numbers per months continuation of breastfeeding (duration), and the increase or decrease (%) in log transformed cell numbers of groups exposed to breastfeeding relative to the reference group (never breastfeeding). Missing in in categories 0-6-9 months (n = 14 at 25 months, n = 46 at 6 years). Breastfed groups include both partially and exclusively breastfed children. Adjusted for maternal education and maternal alcohol use during pregnancy. *P-value <0.05.
(DOC)

S3 Table Antibody panel used for 6-color flow cytometry. FITC = fluorescein isothiocyanate, PE = phycoerythrin, PerCPCy5.5 = peridin chlorophyll protein, PE-Cy7 = phycoerythrin-cyanin dye, APC = allophycocyanin and APC-Cy7 = allophycocyanin-cyanin dye, poly = polyclonal antibody.
(DOC)

S1 Fig Summarizing mechanism of how breastfeeding might affect adaptive memory. In absence of breast milk, the infant’s B and T cells respond to microorganisms in the intestine and generate long-lived memory cells and IgA (blue) that circulate through the body (left). Breast milk contains immune modulating components (right). Of these, maternal sIgA (green) is able to catch microorganisms and prevent recognition of these by B-cells. This might inhibit B-cell responses and B-cell memory formation. Other immunostimulatory components, such as exosomes, might stimulate naive T cells and increase T-cell memory formation. Abbreviations: Bn, naïve B cell; Bm, memory B cell; DC, dendritic cell; pc,plasma cell; Tn, naive T cell; Tm, memory T cell.
(EPS)
