# Supplementary material for: Decreased Memory B Cells and Increased CD8 Memory T Cells in Blood of Breastfed Children: The Generation R Study
Source: PLoS One. 2015 May 18;10(5):e0126019. doi: 10.1371/journal.pone.0126019 (PMC4436360; doi:10.1371/journal.pone.0126019)
Supplement: S1 Table — Values are means (SD), absolute numbers (percentages) or #medians (90% range). * Significantly different between breastfeeding and no breastfeeding groups. (DOC) [file pone.0126019.s001.doc]

**Decreased Memory B cells and Increased CD8 Memory T cells in Blood of Breastfed Children: The Generation R study**

**Running title:** Impact of breastfeeding on adaptive immunity

Michelle A.E. Jansen, ^1,2,3^ Diana van den Heuvel, ^3^ Menno C. van Zelm, ^3^ Vincent W.V. Jaddoe, ^1,2,4^ Albert Hofman, ^4^ Johan C. de Jongste, ^2^ Herbert Hooijkaas,^3^ Henriette A. Moll ^2^

**Affiliations:**

^1^The Generation R Study Group, Erasmus MC, University Medical Center, Rotterdam, the Netherlands, ^2^the Department of Pediatrics, Sophia Children’s Hospital, Erasmus MC, University Medical Center, Rotterdam, the Netherlands, ^3^the Department of Immunology, Erasmus MC, University Medical Center, Rotterdam, the Netherlands and ^4^the Department of Epidemiology, Erasmus MC, University Medical Center, Rotterdam, the Netherlands.

**S1 Table. Maternal and infant characteristics of the study population at 14, 25 months and 6 years**

|  | | **14 months (n=166)** | | **25 months (n=112)** | | **6 years (n=332)** | |
| --- | --- | --- | --- | --- | --- | --- | --- |
|  | | **No breastfed (n=16)** | **Breastfed**  **(n=150)** | **No breastfed (n=16)** | **Breastfed**  **(n=96)** | **No breastfed**  **(n=37)** | **Breastfed**  **(n=295)** |
| **Maternal characteristics** | |  | | | | | |
| Age (Mean + SD; years) | | 31 (4.2) | 32 (3.9) | 31 (4.1) | 32 (3.8) | 31 (3.6) | 32 (3.6) |
| **Educational level (n; %)** | |  |  |  |  |  |  |
|  | **Lower** | **7 (44%)** | **59 (40%)** | **12 (75%)** | **31 (33%)** | **22 (59%)** | **96 (33%)** |
|  | **Higher** | **9 (56%)** | **90 (60%)** | **4 (25%)** | **64 (67%)*** | **15 (41%)** | **196 (67%)*** |
| Net household income per month (n; %) | |  |  |  |  |  |  |
|  | < € 2400 | 0 (0%) | 21 (15%) | 1 (8%) | 13 (15%) | 4 (12%) | 33 (13%) |
|  | > € 2400 | 13 (100%) | 112 (84%) | 12 (92%) | 73 (85%) | 30 (88%) | 227 (87%) |
| Smoking continued during pregnancy (n; %) | | 2 (14%) | 16 (15%) | 1 (7%) | 8 (11%) | 6 (20%) | 32 (15%) |
| Alcohol use continued during pregnancy (n; %) | | 6 (43%) | 41 (38%) | 4 (29%) | 29 (39%) | 11 (37%) | 83 (37%) |
| Body Mass Index before pregnancy (Mean + SD; kg/m^2^) | | 25 (5) | 24 (5) | 25 (5) | 23 (4) | 24 (5) | 24 (4) |
| Fever in third trimester of pregnancy (n; %) | | 1(6%) | 9 (6%) | 2 (13%) | 5 (6%) | 2 (6%) | 19 (7%) |
| Maternal atopy (eczema, allergy HDM, hay-fever)(n; %) | | 3 (21%) | 51 (36%) | 6 (40%) | 41 (46%) | 9 (27%) | 109 (40%) |
| Paternal atopy (eczema, allergy HDM, hay-fever) (n; %) | | 3 (23%) | 41 (29%) | 5 (39%) | 24 (28%) | 13 (39%) | 77 (29%) |
| Family history of asthma / atopy (n; %) | | 5 (31%) | 77 (52%) | 9 (56%) | 56 (59%) | 20 (54%) | 156 (54%) |
| Any reported autoimmune disease (diabetes mellitus, SLE, arthritis, MS, thyroid disorder, or celiac disease) (n; %) | | 0 (0%) | 3 (2%) | 0 (0%) | 2 (2%) | 2 (5%) | 8 (3%) |
| Mode of delivery (n; %) | |  |  |  |  |  |  |
|  | Vaginal | 9 (56%) | 19 (64%) | 7 (47%) | 48 (55%) | 20 (59%) | 182 (65%) |
|  | Forceps or vacuum assisted | 2 (13%) | 30 (21%) | 2 (13%) | 21 (24%) | 5 (15%) | 59 (21%) |
|  | Caesarian section | 5( 31%) | 21 (15%) | 6 (40%) | 19 (22%) | 9 (26%) | 40 (14%) |
| Premature rupture of membranes (n; %) | | 1 (6%) | 2 (1%) | 1 (6%) | 2 (2%) | 3 (8%) | 4 (2%) |
| **Infant characteristics (n=258)** | |  | | | | | |
| Males (n; %) | | 9 (56%) | 77 (51%) | 7 (44%) | 47 (49%) | 20 (54%) | 144 (49%) |
| Gestational age ( Mean + SD; weeks) | | 39.2 (2.2) | 40.1 (1.9) | 39.7 (1.9) | 39.9(1.5) | 39.7 (1.7) | 40.1 (1.6) |
| Preterm birth (<37 weeks) (n; %) | | 1 (6% | 3 (2%) | 1 (6%) | 4 (4%) | 2 (5%) | 8 (3%) |
| Birth weight ( Mean + SD; grams) | | 3,455 (738) | 3,505 (513) | 3,516 (614) | 3,542 (480) | 3,543 (554) | 3,531 (508) |
| Apgar score at 5 min <7 (n; %) | | 0 (0%) | 3 (2%) | 0 (0%) | 0 (0%) | 0 (0%) | 3 (1%) |
| Birth season (n; %) | |  |  |  |  |  |  |
|  | Winter (Dec-Jan-Feb) | 2 (13%) | 38 (25%) | 3 (19%) | 28 (29%) | 6 (16%) | 73 (25%) |
|  | Spring (Mar-Apr-May) | 5 (31%) | 41 (27%) | 3 (19%) | 26 (27%) | 6 (16%) | 93 (31%) |
|  | Summer (Jun-Jul-Aug) | 3 (18%) | 32 (21%) | 4 (25%) | 21 (22%) | 11 (30%) | 65 (22%) |
|  | Autumn (Sept-Oct-Nov) | 6 (38%) | 39 (26) | 6 (37%) | 21 (22%) | 14 (38%) | 64 (22%) |
| Siblings >1 (n; %) | | 2 (12%) | 17 (11%) | 1 (6%) | 8 (8%) | 2 (5%) | 26 (9%) |
| Day-care >16 hours /week (n; %) | | 3 (33%) | 51 (47%) | 4 (25%) | 33 (34%) | 5 (33%) | 99 (47%) |
| Fever in first 6 months (n; %) | | 7 (78%) | 80 (63%) | 3 (60%) | 51 (62%) | 13 (57%) | 175 (67%) |
| Age at focus visit (Median + range; months/years) | | 14.4  (13.2-16.3) | 14.4  (13.1-17.4) | 25.3  (23.4-27.5) | 25.2  (23.3-29.8) | 6.0  (5.8-6.6) | 5.8  (5.1-7.2) |

Values are means (SD), absolute numbers (percentages) or ^#^medians (90% range). * Significantly different between breastfeeding and no breastfeeding groups
