# Supplementary material for: Decreased Memory B Cells and Increased CD8 Memory T Cells in Blood of Breastfed Children: The Generation R Study
Source: PLoS One. 2015 May 18;10(5):e0126019. doi: 10.1371/journal.pone.0126019 (PMC4436360; doi:10.1371/journal.pone.0126019)
Supplement: S2 Table — Values are log transformed regression coefficients (95% confidence interval of cell numbers (*10e9/L)) derived from linear regression models and reflect the increase or decrease (%) in log transformed cell numbers per months continuation of breastfeeding (duration), and the increase or decrease (%) in log transformed cell numbers of groups exposed to breastfeeding relative to the reference group (never breastfeeding). Missing in in categories 0-6-9 months (n = 14 at 25 months, n = 46 at 6 years). Breastfed groups include both partially and exclusively breastfed children. Adjusted for maternal education and maternal alcohol use during pregnancy. *P-value <0.05. (DOC) [file pone.0126019.s002.doc]

**Decreased Memory B cells and Increased CD8 Memory T cells in Blood of Breastfed Children: The Generation R study**

**Running title:** Impact of breastfeeding on adaptive immunity

Michelle A.E. Jansen, ^1,2,3^ Diana van den Heuvel, ^3^ Menno C. van Zelm, ^3^ Vincent W.V. Jaddoe, ^1,2,4^ Albert Hofman, ^4^ Johan C. de Jongste, ^2^ Herbert Hooijkaas,^3^ Henriette A. Moll ^2^

**Affiliations:**

^1^The Generation R Study Group, Erasmus MC, University Medical Center, Rotterdam, the Netherlands, ^2^the Department of Pediatrics, Sophia Children’s Hospital, Erasmus MC, University Medical Center, Rotterdam, the Netherlands, ^3^the Department of Immunology, Erasmus MC, University Medical Center, Rotterdam, the Netherlands and ^4^the Department of Epidemiology, Erasmus MC, University Medical Center, Rotterdam, the Netherlands.

**S2 Table. Adjusted association between breastfeeding and cell numbers at 25 months and 6 years**

| **Logtransformed cell numbers (*10e9/L)** | | | | | | | | | |
| --- | --- | --- | --- | --- | --- | --- | --- | --- | --- |
| **25 months (n=112)** | | | | | | **6 years (n=332)** | | | |
|  | **B** | **NK** | **T** | **CD4** | **CD8** |  | **B** | **NK** | **T** |
| **DURATION** |  |  |  |  |  | **DURATION** |  |  |  |
| Breastfeeding (n=96)  increase per month | -1 (-4,1) | 0 (-3,3) | 0 (-2,2) | 1 (-2,3) | -2 (-4,1) | Breastfeeding (n=295)  increase per month | -1(-2,1) | 0(-2,1) | 1(-1,2) |
| **EXPOSURE** |  |  |  |  |  | **EXPOSURE** |  |  |  |
| Never (n= 16) | REF | REF | REF | REF | REF | Never (n=37) | REF | REF | REF |
| < 3 months (n=29) | 14 (-10,38) | 27 (-3, 58) | 14 (-7,35) | 11 (-10,32) | 18 (-9,45) | < 3 months (n=112) | -6(-19,8) | 11(-5,26) | 1(-10,12) |
| > 3 < 6 months (n=30) | 0 (-23,23) | 7 (-23, 37) | -2 (-22,18) | -4 (-24,17) | 1 (-25,27) | > 3 < 6 months (n=76) | -5(-19,10) | 7(-10,24) | 2(-10,14) |
| > 6 months (n=37) | 11 (-14,35) | 11 (-20,42) | 12 (-9,33) | 18 (-3,30) | -3 (-30, 24) | > 6 months (n=107) | -12(-26,3) | -1(-17,16) | 3(-8,15) |
| Never (n=15) | REF | REF | REF | REF | REF | Never (n=52) | REF | REF | REF |
| < 6 months (n=50) | -17 (-35, 2) | 3 (-22,27) | -7 (-23,10) | -11 (-28,6) | 3 (-18,25) | < 6 months (n=150) | 3(-8,13) | 6(-6,18) | 1(-8,10) |
| 6-9 months (n=18) | -4 (-29,21) | 1 (-33,34) | -2 (-24,21) | 0 (-23,23) | -2 (-31,28) | 6-9 months (n=50) | 5(-10,20) | 2(-15,19) | 1(-11,13) |
| > 9 months (n=15) | 3 (-26, 32) | -17 (-55, 21) | 9 (-17,35) | 10 (-17,36) | 8 (-26,41) | > 9 months (n=34) | -3(-20,14 | -6(-25,14) | 1(-13,15) |

Values are log transformed regression coefficients (95% confidence interval of cell numbers (*10^e9^/L)) derived from linear regression models and reflect the increase or decrease (%) in log transformed cell numbers per months continuation of breastfeeding (duration), and the increase or decrease (%) in log transformed cell numbers of groups exposed to breastfeeding relative to the reference group (never breastfeeding). Missing in in categories 0-6-9 months (n=14 at 25 months, n=46 at 6 years) Adjusted for maternal education and maternal alcohol use during pregnancy. *P-value <0.05.
