# Supplementary material for: Decreased Memory B Cells and Increased CD8 Memory T Cells in Blood of Breastfed Children: The Generation R Study
Source: PLoS One. 2015 May 18;10(5):e0126019. doi: 10.1371/journal.pone.0126019 (PMC4436360; doi:10.1371/journal.pone.0126019)
Supplement: S3 Table — FITC = fluorescein isothiocyanate, PE = phycoerythrin, PerCPCy5.5 = peridin chlorophyll protein, PE-Cy7 = phycoerythrin-cyanin dye, APC = allophycocyanin and APC-Cy7 = allophycocyanin-cyanin dye, poly = polyclonal antibody. (DOC) [file pone.0126019.s003.doc]

**Decreased Memory B cells and Increased CD8 Memory T cells in Blood of Breastfed Children: The Generation R study**

**Running title:** Impact of breastfeeding on adaptive immunity

Michelle A.E. Jansen, ^1,2,3^ Diana van den Heuvel, ^3^ Menno C. van Zelm, ^3^ Vincent W.V. Jaddoe, ^1,2,4^ Albert Hofman, ^4^ Johan C. de Jongste, ^2^ Herbert Hooijkaas,^3^ Henriette A. Moll ^2^

**Affiliations:**

^1^The Generation R Study Group, Erasmus MC, University Medical Center, Rotterdam, the Netherlands, ^2^the Department of Pediatrics, Sophia Children’s Hospital, Erasmus MC, University Medical Center, Rotterdam, the Netherlands, ^3^the Department of Immunology, Erasmus MC, University Medical Center, Rotterdam, the Netherlands and ^4^the Department of Epidemiology, Erasmus MC, University Medical Center, Rotterdam, the Netherlands.

**S3 Table. Antibody panel used for 6-color flow cytometry**

| **Labeling** | **Conjugated monoclonal antibodies (clone)** | | | | | |
| --- | --- | --- | --- | --- | --- | --- |
|  | **FITC** | **PE** | **PerCP-Cy5.5** | **PE-Cy7** | **APC** | **APC-Cy7** |
| 1 | CD3 | CD16.56 | CD45 | CD4 | CD19 | CD8 |
|  | (SK7) | (B73.1C5.9) | (2D1) | (SK3) | (SJ25C1) | (SK1) |
| 2 | IgD | CD23 | CD19 | CD21 | IgM | CD27 |
|  | (poly) | (EBVCS5) | (SJ25C1) | (B-ly-4) | (G20-127) | (M-T271) |
| 3 | IgA | IgG | CD19 | CD40 | IgM | CD27 |
|  | (poly) | (poly) | (SJ25C1) | (5C3) | (G20-127) | (M-T271) |
| 4 | CD28 | CD197 | CD3 | CD8 | CD45RO | CD27 |
|  | (CD28.2) | (3D13) | (SK7) | (SK1) | (UCHL-1) | (M-T271) |

FITC= fluorescein isothiocyanate, PE=phycoerythrin, PerCPCy5.5= peridin chlorophyll protein,

PE-Cy7= phycoerythrin-cyanin dye, APC=allophycocyanin and APC-Cy7= allophycocyanin-cyanin dye, poly = polyclonal antibody
